# Reducing work pressure and IT problems and facilitating IT integration and audit & feedback help adherence to perioperative safety guidelines: a survey among 95 perioperative professionals

**DOI:** 10.1186/s43058-020-00037-1

**Published:** 2020-05-27

**Authors:** Yvette E. J. J. M. Emond, André P. Wolff, Yvonne A. S. Peters, Gerrit J. A. Bloo, Gert P. Westert, Johan Damen, Hiske Calsbeek, Hub C. Wollersheim

**Affiliations:** 1 Radboudumc, Radboud Institute for Health Sciences, IQ healthcare, Nijmegen, The Netherlands; 2 Radboudumc, Radboud Institute for Health Sciences, Department of Anesthesiology, Pain and Palliative Care, Nijmegen, The Netherlands; 3 Radboudumc, IQ healthcare, PO Box 9101, 114 IQ healthcare, 6500 HB Nijmegen, The Netherlands; 4 University Medical Center Groningen, University of Groningen, Department of Anesthesiology, Pain Center, Groningen, The Netherlands

**Keywords:** Guideline adherence, Implementation, Implementation barriers, Implementation facilitators, Patient safety, Perioperative care

## Abstract

**Background:**

To improve perioperative patient safety, guidelines for the preoperative, peroperative, and postoperative phase were introduced in the Netherlands between 2010 and 2013. To help the implementation of these guidelines, we aimed to get a better understanding of the barriers and drivers of perioperative guideline adherence and to explore what can be learned for future implementation projects in complex organizations.

**Methods:**

We developed a questionnaire survey based on the theoretical framework of Van Sluisveld et al. for classifying barriers and facilitators. The questionnaire contained 57 statements derived from (a) an instrument for measuring determinants of innovations by the Dutch Organization for Applied Scientific Research, (b) interviews with quality and safety policy officers and perioperative professionals, and (c) a publication of Cabana et al. The target group consisted of 232 perioperative professionals in nine hospitals. In addition to rating the statements on a five-point Likert scale (which were classified into the seven categories of the framework: factors relating to the intervention, society, implementation, organization, professional, patients, and social factors), respondents were invited to rank their three most important barriers in a separate, extra open-ended question.

**Results:**

Ninety-five professionals (41%) completed the questionnaire. Fifteen statements (26%) were considered to be barriers, relating to social factors (*N* = 5), the organization (*N* = 4), the professional (*N* = 4), the patient (*N* = 1), and the intervention (*N* = 1). An integrated information system was considered an important facilitator (70.4%) as well as audit and feedback (41.8%). The Barriers Top-3 question resulted in 75 different barriers in nearly all categories. The most frequently reported barriers were as follows: time pressure (16% of the total number of barriers), emergency patients (8%), inefficient IT structure (4%), and workload (3%).

**Conclusions:**

We identified a wide range of barriers that are believed to hinder the use of the perioperative safety guidelines, while an integrated information system and local data collection and feedback will also be necessary to engage perioperative teams. These barriers need to be locally prioritized and addressed by tailored implementation strategies. These results may also be of relevance for guideline implementation in general in complex organizations.

**Trial registration:**

Dutch Trial Registry: NTR3568.

Contributions to the literature

- The perioperative setting is a relatively unexplored area with respect to guideline implementation. The determinants for the implementation of the perioperative safety guidelines have not been investigated before.
- Knowledge on barriers and drivers of perioperative guideline adherence can be used to improve perioperative patient safety.
- Our study contributes to gaps in literature and provides a better understanding of real-world guideline implementation barriers and drivers among target users within the perioperative care process. This is important for improving the use of the perioperative guidelines in all (Dutch) hospitals.
- The findings may also be generalized to the implementation of other guidelines, in other complex areas of healthcare.

## Background

Rising demands for efficiency, effectiveness, and safety with increasingly limited resources put healthcare systems worldwide under pressure to continuously deliver high-quality and safe care [1, 2]. It is recognized that implementation of best practices in healthcare is slow, incomplete, and often not sustainable, with variations in compliance [3].

In 2008, the World Health Organization (WHO) Surgical Safety Checklist was introduced to ensure the consistent use of safety processes [4]. The application of the WHO checklist has led to reductions in adverse events [5] and cost savings [6]. In the Netherlands, three novel guidelines with recommendations for a safe preoperative, peroperative, and postoperative care pathway including Patient Safety Indicators (PSIs) (see Additional file 1) were published [7–10]. These recommendations involve the process and organization of perioperative care, instead of the clinical content of the care process, with descriptions of responsibilities and standardization of methods to reduce risks related to—among others—handovers, wrong-site surgery, infection prevention, allergies, retained surgical clamps or sponges, and blood-clotting problems. In addition, several stop moments, i.e., stopping rules translated into a checklist, have been introduced, such as the preoperative screening, the time-out, and the sign-out, in order to check whether agreements have been met and a patient can go to the next phase in the care process (all stop moments in the perioperative trajectory have to be performed in each surgical patient). The goal of the guideline initiative is a culture that demonstrably improves perioperative outcomes by the timely administration of antibiotic prophylaxis where indicated and the performance of the entire STOP bundle (i.e., a composite outcome defined as the percentage of patients in which all the stop moments in the whole perioperative care process have been performed) in all patients.

Implementation of these perioperative safety guidelines is expected to reduce adverse patient outcomes. Several studies showed that using stop moments and the timely administration of antibiotic prophylaxis improve perioperative outcomes [5, 11, 12]. Adherence to these guidelines has, however, shown to be far from optimal [10]. These results clearly show that a thorough analysis of the barriers and drivers for adherence to the perioperative safety guidelines is essential to further improve perioperative patient safety.

One of the most consistent findings in healthcare services research is the gap between best practice on the one hand and the actual clinical care on the other. Despite the considerable efforts in developing and implementing evidence-based guidelines, only a modest impact has been found on clinical practice [13–17]. Producing change is usually less easy, particularly when multiple medical and paramedical disciplines are involved or the innovation requires complex changes in clinical practice, better collaboration between disciplines, or changes in the organization of care. A comprehensive study in the USA showed that only about half of the patients (55%) received recommended care as described in the guidelines [18]. In addition, it has been demonstrated that changing physicians’ behavior is extremely difficult [19]. From a psychological perspective, this is called the “knowledge-behavior gap,” indicating the difference between what we know we should do and what we actually do in clinical daily practice [3, 19]. Reflecting on this failure of implementation, most experts in healthcare improvement now emphasize the crucial importance of acquiring a good understanding of the problem, the target group, its setting, and the obstacles to change [20]. To bridge the gap between desired and current patient care, we need an in-depth understanding of the barriers and incentives to achieve change in practice. In general, little is known about the processes and factors responsible for how healthcare professionals change their practice methods when they have to adhere to a new guideline and do something they did not do before.

Many factors may influence the implementation of the perioperative safety guidelines in practice. In scientific research on determinants of change, it is important to obtain a comprehensive insight into these factors. A theoretical approach can help explain these factors and possibly help target interventions. To answer our research question and structure the analysis, we used a framework of Van Sluisveld et al. [21] that provides insight into the process of the implementation of scientific evidence, as well as into factors influencing this process. This framework is based on three models related to implementing change: the implementation of change model of Grol and Wensing [22, 23], the framework of knowledge–attitude–behavior-related barriers for guideline adherence of Cabana et al. [19], and the framework for adherence to clinical practice guidelines in the intensive care unit (ICU) of Cahill et al. [24]. Measuring determinants is thus a first and basic activity for all researchers and implementation supervisors. The Dutch Organization for Applied Scientific Research (TNO) has developed a short diagnostic tool for this [25]. The TNO has been conducting research since 1999 into determinants that predict the actual use of innovations. In 2012, the organization performed a meta-analysis to arrive at an empirically supported list of 29 determinants, which has been converted into a generic diagnostic tool: the Measurement Instrument for Determinants of Innovations (MIDI). Based on the literature [21, 23], the following seven categories of factors can be identified: features of the innovation itself (e.g., complexity, credibility, amount of information, feasibility), features of the societal context (e.g., political developments and policies, legal obligations, and regulations), features of the interventions used for implementation and dissemination (e.g., exposure to implementation efforts), features of the institutional context (e.g., organizational structure, organization of care and logistic processes, time, [qualified] staff, task divisions, [financial] resources, equipment, IT structure), features of the social setting (e.g., behavior of colleagues, collaboration, culture in the team), features of the target group of professionals who should use the innovation (e.g., their awareness, knowledge, motivation, opinions, attitudes, behavioral routines, habits, expectations), and features of the patients (e.g., their capacities, preferences) [21, 23].

Tailored and barrier-driven implementation strategies are needed to improve adherence to guidelines in practice. An adequate analysis of barriers and facilitators to the implementation of a guideline is considered to be a first and important step in improving guideline adherence and subsequently quality and safety of care [3, 20]. The barriers and facilitators identified can be used to develop tailored implementation strategies. Systematic tailoring entails (at least) three key steps: identification of the determinants of practice (i.e., barriers and facilitators), designing implementation interventions appropriate to the determinants, and application (and evaluation) of implementation interventions that are matched to the identified determinants [26]. In this study, relevant barriers and drivers for the implementation of the perioperative safety guidelines and more specifically the stop moments were explored and prioritized, aiming to offer opportunities to develop determinant-driven and tailored interventions to improve the implementation and use of the perioperative guidelines in practice. Some of the determinants will be generic (they influence many implementation processes), while others will be specific to the guidelines being implemented. We also acknowledged the role of the context in implementation. The factors that determine whether the implementation of an innovation is successful or not are many and varied. Factors may be connected to the setting in which the innovation is to be implemented. Each target group or setting is, in some sense, unique. There are often clear differences between groups of care providers and hospitals with respect to their barriers and drivers for change. Effective implementation, therefore, cannot take place without an analysis of the setting and the target group in which the implementation is to take place. We, therefore, also performed subgroup analyses as in the study of Van Sluisveld et al. [27].

## Methods

### Study design

An anonymous online questionnaire survey about factors that may influence the implementation of the perioperative safety guidelines was conducted among 46 anesthesiologists, 33 surgeons, 124 nurses (42 operation room (OR), 34 anesthesia, 23 recovery, and 25 ward), and 29 ICU employees in all nine hospitals enrolled in the IMPROVE (IMPlementatie Richtlijnen Operatieve VEiligheid (Implementation of Perioperative Safety Guidelines)) study [28] (two academic, four tertiary teaching, and three regional hospitals). We asked the contact persons to provide the contact data of at least one respondent per discipline. Because of the expected low respondence rates, we used an information-oriented selection strategy to maximize the utility of information from single cases or small samples. The respondents were purposively selected as key professionals by the contact persons in their hospitals on the basis of relevance (expectations about their information content). After pilot testing the questionnaire within the IMPROVE research team for comprehensibility, completeness, overlapping questions, and length, the questionnaire was sent by e-mail, followed by two reminder e-mails after 1 and 3 weeks, respectively. Informed consent was implied by completion and return of the questionnaire.

### Questionnaire

The questionnaire was self-developed, based on the theoretical framework for classifying barriers and facilitators as described by Van Sluisveld et al. [21]. According to this framework, barriers and facilitators are grouped according to whether they are related to the intervention, society, implementation, organization, professional, patient, or social factors. The content was derived from the instrument for measuring determinants of innovations by TNO [25], from the publication of Cabana et al. [19] about why physicians do not follow clinical practice guidelines, and from the results of 19 individual semi-structured interviews with quality and safety policy officers and perioperative key professionals in five Dutch hospitals. These interviews were held during the development process of the PSIs belonging to the perioperative safety guidelines [28]. The interviews were audio recorded over the phone. Interviewees were asked, by means of a topic guide, about the factors that hindered or aided the guideline implementation process.

The final questionnaire covered six sections: (1) demographic and professional characteristics; (2) guideline adherence topics: the intervention characteristics, the societal context, the institutional characteristics, the social context, the professional characteristics, and the patient characteristics; (3) adherence to the stop moments; (4) local implementation; (5) prioritizing barriers into a personal top-3 list; and (6) wishes and needs with respect to the future implementation of the guidelines. Respondents were asked to score the statements on a five-point Likert scale or to answer open-ended questions on their opinions (e.g. “how can the implementation be improved?”) and ranking their three most important barriers. The professionals were also asked to score the motivation or readiness within their discipline to work or start working according to the perioperative guidelines on a 10-point scale (1 = my discipline is not motivated nor willing to work according to the perioperative guidelines and to perform the stop moments; 10 = my discipline is fully motivated and willing to work according to the perioperative guidelines and to perform the stop moments).

### Data analysis

The questionnaire results were analyzed using SPSS 20. In case of incomplete surveys, we applied pairwise deletion of missing values. Thus, for each analysis, questionnaires were excluded from the analysis only if the concerning variables could not be derived from the questionnaire due to missing data in that questionnaire. Descriptive statistics were used (percentages, mean, standard deviation). We first recoded the statements that were negatively formulated to match those positively formulated (so that a higher score indicated a positive response). We then grouped the statement scores 1 and 2, indicating a negative response (disagreement with the statement), as well as the scores 4 and 5, indicating a positive response (agreement with the statement). The score 3 indicated a neutral response. We sorted the statements in a table, in which 100% was the highest (i.e., all respondents scored the statement as positive) and 0% was the lowest measure of agreement. We considered a statement to reflect a perceived barrier if fewer than 50% of the respondents scored the statement as positive (score 4 or 5).

To explore whether there were differences in answers with regard to the statements between subgroups based on discipline and hospital type, we conducted subgroup analyses, i.e., Student’s *t* tests with a *p* value < 0.05 as threshold to signal differences.

## Results

### Characteristics of the respondents

We received 95 completed questionnaires, resulting in a response rate of 41%. Thirty-two percent of the questionnaires were incomplete, with one or more questions missing. There was no indication that “sensitive” questions were avoided. Questionnaires were deleted from the analysis only if the required question was missing. This resulted in analyses of 93–99% of the questionnaires. The majority of the professionals were female (56%). Respondents had 18.6 ± 9.9 years (range 1–40 years) of work experience in their current function and 15.8 ± 10.7 years (range 1–42 years) of work experience in their current hospital. All respondents were contracted to only one of the included hospitals, with 80% affiliated to non-academic hospitals. Eight percent had a function other than the prespecified professions (see the “Study design” section), e.g., OR executive; operational executive; head of the OR; surgical team, OR team, or nursing ward team leader. Response was lowest among ward nurses (response rate of 20%), OR nurses (28%), and ICU employees (34%). The response of the other disciplines was > 41%. Table 1 summarizes the demographic and professional characteristics of the responding professionals.

**Table 1:** Demographic and professional characteristics of the responding perioperative key professionals (*N* = 95)

Gender, *N* (%)	
Male	41 (43.6)
Female	53 (56.4)
Years clinical experience in the current specialty, mean (SD)	18.61 (9.87)
Years clinical experience in the current specialty within the current hospital, mean (SD)	15.83 (10.74)
Job title, *N* (%)	
Anesthesiologist	20 (21.3)
Surgeon	14 (14.9)
Anesthesia nurse	14 (14.9)
OR nurse	12 (12.8)
Recovery nurse	11 (11.7)
ICU employee	10 (10.6)
Ward nurse	5 (5.3)
Otherwise	8 (8.5)
Type of hospital, *N* (%)	
Academic hospital (*N* = 2)	19 (20.0)
Tertiary teaching hospital (*N* = 4)	51 (53.7)
Peripheral hospital (*N* = 3)	25 (26.3)

### Adherence to the stop moments

Sixty-five percent of the respondents reported that the performance of the stop moments was fully integrated in the daily routines of the perioperative process within their hospital. According to 69% of the respondents, stop moments were sometimes skipped.

### Perceived barriers

Fifteen of the 57 statements (26.3%) were considered to be barriers (Table 2). These had to do with the following:

1. Social context: Respondents did not care about the opinion of their colleagues; difficulty in bringing the entire team together to carry out a stop moment; colleagues did not always set a good example; the experience of (social) pressure to work not according to the perioperative guidelines; and the impression that their hospital has no open culture, in which everyone should dare to speak up in a social atmosphere of perceived safety
2. Organizational characteristics: the perception that no measures were taken by their hospital to ensure that new employees are adequately instructed in the application of the perioperative guidelines; a lack of financial resources and insufficient time to integrate the perioperative guidelines in their daily work; and no evaluation of the results of working according to the perioperative guidelines in terms of patient safety (e.g., mortality or complications)
3. Professional characteristics: the perception of a safety problem in their hospital; no clear opinions on the distribution of tasks and responsibilities with regard to perioperative care; the perception that working according to the guidelines takes a lot of time (delaying the workflow), which goes at the expense of production and leads to increased work pressure
4. Patient characteristics: respondents thought that patients did not expect them to apply the perioperative guidelines
5. Intervention characteristics: difficulty to adjust daily routines accordingly to the guidelines

**Table 2:** Results of the statements on the determinants of guideline adherence (*N* = 57)

Category	Statement on perceived barriers	Agree %	Disagree %	B
S	My direct supervisors expect me to apply the perioperative guidelines.	91.8	0.0	
O	Working according to the guidelines is checked in my hospital.	89.8	10.2	
P	I find it important to improve patient safety with the perioperative guidelines.	85.7	3.1	
P	I have the knowledge to use the perioperative guidelines.	83.8	4.0	
I	The guidelines clearly indicate which activities I have to perform and in which order.	81.0	4.0	
S	The majority to (almost) all healthcare providers in my hospital really work according to the perioperative guidelines.	79.1	7.7	
S	My colleagues expect me to apply the perioperative guidelines.	78.6	2.0	
I	I feel (very) positive about guidelines in general.	78.3	3.3	
I	I (totally) agree to the content of the guidelines.^a^	78.0	8.0	
S	I can count on sufficient support and involvement from the management by applying the perioperative guidelines.	78.0	3.0	
P	My hospital puts a lot of efforts into the improvement of patient safety along the implementation of the perioperative guidelines.	76.2	1.0	
S	There is enough support in my discipline to work according to the perioperative guidelines.	75.8	4.0	
P	I expect patient safety to increase with the perioperative guidelines.	75.8	6.1	
Sy	The fact that safety stands high on the social agenda is for me (definitely) of influence on the use of the perioperative guidelines.^b^	74.7	10.1	
S	When it comes to the use of the perioperative guidelines, I care about my direct supervisor’s opinion.	73.2	5.2	
I	I find the guidelines easy to use.	71.1	9.3	
IP	There are enough interventions undertaken to put the perioperative guidelines on the hospital agenda.	71.1	27.8	
IP	It is clear to me who is in charge with respect to the implementation of the perioperative guidelines in my department.	70.5	25.0	
I	The perioperative guidelines are based on sound and sufficient evidence.^a^	70.4	7.1	
Sy	A visit by the Inspectorate affects working according to the perioperative guidelines.	69.4	9.2	
S	The cooperation with other disciplines or between departments regarding the execution of the perioperative guidelines is (very) good.	68.1	1.1	
P	The perioperative guidelines match with current or previous hospital initiatives related to patient safety.	67.0	0.0	
IP	There is a perioperative opinion leader within my discipline.^b^	65.2	34.8	
IP	My hospital pays a lot of attention to the implementation of the perioperative guidelines.	63.6	4.0	
I	The perioperative guidelines leave enough room for personal interpretation and adaptation to the specific needs of the department and discipline.^a^	63.0	23.9	
I	The perioperative guidelines provide all the information and materials needed to work with them.	62.6	11.1	
P	I am able to follow the guidelines, even when I am busy or my colleagues do not comply (*i.e.* in stressful circumstances).	62.5	2.1	
S	My direct supervisors set a good example by working according to the guidelines.	62.2	1.0	
I	The guidelines fit well with how I was used to work before.	61.9	11.3	
P	Working according to the perioperative guidelines is advantageous.^a^	61.5	5.5	
PA	When it comes to the use of the perioperative guidelines, I care about the opinion of my patients.	61.5	13.5	
IP	I feel sufficiently involved into the implementation of the perioperative guidelines within my hospital.	61.2	14.3	
P	Working according to the perioperative guidelines does not take a lot of time at the expense of the patients.	60.2	10.2	
P	Working according to the guidelines does not affect my clinical freedom and autonomy.	60.0	10.5	
O	I have easy access to information about the use of perioperative guidelines in my hospital.	59.0	15.0	
O	I receive sufficient materials and facilities to be able to use the perioperative guidelines as intended.	58.8	13.4	
O	In my hospital there is sufficient manpower to be able to use the perioperative guidelines as intended.	56.7	12.4	
O	Steps are taken in case of not working according to the perioperative guidelines.	56.2	43.8	
O	I receive regular feedback about perioperative incidents and complications from my hospital.^b^	56.1	13.3	
P	The tasks and responsibilities regarding the perioperative guidelines are known to all employees.	54.1	18.4	
I	The effects of using the guidelines are clearly visible.	51.0	19.8	
S	Everyone takes their responsibility when it comes to working safely; thus, actually addressing each other in unsafe situations and guideline non-adherence.	51.0	15.0	
S	When it comes to the use of the perioperative guidelines, I care a lot about the opinion of my colleagues.	49.0	18.4	V
O	Steps are taken in my hospital so that new employees get sufficiently trained in the use of the perioperative guidelines.^b^	47.4	12.4	V
PA	Patients definitely expect me to apply the perioperative guidelines.	46.4	7.2	V
O	In my hospital there are sufficient financial resources available to be able to use the perioperative guidelines as intended.^b^	46.3	9.5	V
S	It is not difficult to bring the entire team together to carry out a stop moment.^b^	45.1	14.3	V
I	I do not find it difficult to adjust my daily routines and way of working according to the guidelines.	44.3	11.4	V
O	The results of working according to the perioperative guidelines in terms of patient safety (*e.g.* mortality or complications) are evaluated.	44.0	56.0	V
S	Colleagues always set a good example by applying the guidelines.	43.9	4.1	V
S	I never experience (social) pressure not to work according to the perioperative guidelines.^a^	39.1	6.5	V
O	My hospital provides me with sufficient time to integrate the perioperative guidelines in my daily work.	36.7	20.4	V
P	There is no perioperative safety problem in my hospital.	35.0	26.0	V
S	In my hospital is an open contact culture, in which everyone dares to speak up to each other about his or her actions and behavior and this is also accepted by everyone.	32.7	33.7	V
P	Everyone agrees with the distribution of tasks and responsibilities with regard to perioperative care.	31.6	13.3	V
P	Working according to the perioperative guidelines does not take a lot of time, at the expense of production.^a^	29.5	25.3	V
P	Working according to the perioperative guidelines does not lead to increased work pressure.^b^	21.1	35.6	V

*I* intervention characteristics, *Sy* societal context, *IP* implementation characteristics, *O* organizational characteristics, *S* social context, *P* professional characteristics, *PA* patient characteristics, *B* barrier

^a^Answer influenced by perioperative discipline

^b^Answer influenced by hospital type

### Local implementation

Sixty-one percent of the respondents participated in the implementation interventions in their hospital. Reasons for not participating were: time pressure (37%); absence during the interventions (35%); no priority (19%); no obligation (11%); doubts about the added value of the interventions (8%); and colleagues did not also participate (3%).

### Perceived facilitators for future implementation

On average, motivation was valued at 7.5 out of 10 (SD = 1.28; range = 4–10).

The majority of health professionals reported that integrated information systems (70.4%) and audit and feedback (41.8%) were the most useful tools to support the appliance of the perioperative guidelines and stop moments. Furthermore, approximately 37% of our respondents indicated that education would improve the implementation of the guidelines, whereas 36% indicated that they would like (digital) checklists. Thirty-three percent wished for more administrative support, and 24% requested the availability of more computers, smartphones, or tablets to fill out the checklists.

In Additional file 2, the answers to the open-ended questions are provided.

### Barriers Top-3

A total of up to 75 unique barriers to effective guideline implementation were found (Table 3). Sixty-nine professionals reported at least one barrier, 54 professionals ranked at least two barriers, and 35 professionals ranked three barriers. Overall, organizational barriers were most frequently reported.

**Table 3:** Barriers for guideline adherence (75 unique barriers) ranked in Barriers Top-3 (*N* = 158 in total)

	Category	Barrier	1st place N (%)	2nd place N (%)	3rd place N (%)
	Intervention characteristics		6 (8.7)	3 (5.6)	1 (2.9)
1		Lack of scientific evidence	2	0	0
2		Complexity—unclear/ambiguous guidelines	2	0	0
3		Complexity—the guidelines are too extensive	1	0	0
4		Risk for the patient	1	0	0
5		Complexity—the guidelines are too detailed	0	1	0
6		Complexity—the guidelines contain many actions to carry out	0	1	0
7		Patient unfriendly guidelines	0	1	0
8		Complexity—the guidelines contain many stop moments	0	0	1
	Societal context		0 (0.0)	0 (0.0)	0 (0.0)
	Implementation characteristics		0 (0.0)	2 (3.7)	0 (0.0)
9		Being exposed too late to implementation efforts	0	2	0
	Organizational characteristics		27 (39.1)	23 (42.6)	14 (40.0)
10		Lack of time/time pressure	13	8	5
11		Workload	4	1	0
12		IT	1	3	2
13		Relevant information is missing, incomplete, or wrong	1	2	1
14		Organizational preconditions—preparations not adequately executed	0	2	0
15		Staff—capacity/lack of personnel	1	0	1
16		Organizational preconditions—a large group has to do the same thing at the same time	1	0	0
17		Staff—turnover of personnel	1	0	0
18		Logistics	1	0	0
19		Availability of resources and materials	1	0	0
20		Impossibility to meet all requirements within the current organization	1	0	0
21		Capacities	1	0	0
22		Organization of care processes	1	0	0
23		Unclear who is responsible for what; in case of shared responsibility, no one feels responsible	0	1	0
24		Organizational complexity	0	1	0
25		Prioritizing	0	1	0
26		Pressure to run production/finish surgeries on time	0	1	0
27		Lack of decisional power	0	1	0
28		Guests not aware of hospital—specific implementation of the guidelines	0	1	0
29		Organizational preconditions—nursing preparations not carried out	0	0	1
30		Too many communication lines	0	0	1
31		Low standard of working procedures	0	0	1
32		Too bureaucratic	0	0	1
	Social context		11 (15.9)	6 (11.1)	3 (8.6)
33		Culture	3	1	0
34		Collaboration (by nurses)	2	0	0
35		It is not accepted that the perioperative process is stopped or slowed down because some earlier stop moments are not (correctly) performed	1	0	0
36		Being overruled by doctors	1	0	0
37		Absence of anesthesiologist during sign-out	1	0	0
38		The surgeon as initiator for performance of the time-out and sign-out	1	0	0
39		Not taken seriously or involved in the TOP by the rest of the team	1	0	0
40		Lack of initiative of the team	1	0	0
41		Lack of support	0	1	1
42		Collaboration by some surgeons	0	1	0
43		Having to appeal people	0	1	0
44		Inefficient teamwork/people have different interests when working in shifts	0	1	0
45		Pressure by surgeons to start the surgery, while the time-out is not listed yet	0	1	0
46		Opposition by colleagues	0	0	1
47		Social pressure	0	0	1
48		Communication problems	0	0	1
	Professional characteristics		9 (13.0)	13 (24.1)	3 (37.1)
49		Attitude (of especially doctors)	2	0	1
50		Opinions—too excessive	2	1	0
51		Behavioral routines	0	2	0
52		Opinions—adherence to the guidelines may create a wrong sense of safety (i.e. hidden unsafety) by weakening independent thinking and responsibility taking	0	2	0
53		Awareness and knowledge about the importance and purpose	0	1	2
54		Behavior	1	1	0
55		Opinions—customization is preferred over standardization	1	0	0
56		Opinions—in some situations it makes totally no sense to apply the guidelines	1	0	0
57		Unclear handwriting	1	0	0
58		Opinions—finding it not useful to repeat things too often	0	1	0
59		Forgetting	0	1	1
60		Consideration for the wishes of the patient/perceiving patient discomfort	0	1	1
61		Concerns about whether the use of checklists promotes a mentality of just ticking boxes	0	1	0
62		Embarrassment toward the patients by asking several times the same question	0	1	0
63		Common sense	0	0	1
64		Motivation	0	0	1
65		Indifferent following of procedures	0	0	1
66		Self-overestimation	0	0	1
67		Personality	0	0	1
68		Lack of interest	0	0	1
69		Rationalities to allow deviant behavior (*e.g.* not following guidelines will not harm patients)	0	0	1
70		Own interpretation	0	0	1
	Patient characteristics		11 (15.9)	4 (7.4)	3 (8.6)
71		Emergency patient	9	2	2
72		Patient ability/cognitive abilities	1	1	0
73		The patient in general	1	0	0
74		Language problems	0	1	0
75		Preferences	0	0	1
	No barriers perceived		5 (7.2)	3 (5.6)	1 (2.9)
	Total		69 (100)	54 (100)	35 (100)

The most frequently mentioned barriers concerning the innovation itself were skepticism with regard to the perioperative safety guidelines, which were considered to lack scientific evidence and to have a complex nature (i.e., unclear and ambiguous). A barrier regarding the implementation of the guidelines was a lack of timeliness, i.e., no early exposure to the implementation efforts. Frequently mentioned organizational barriers were time pressure, IT problems and lacking IT facilities, workload, suboptimal organizational preconditions (e.g., preparations were not adequately performed), and incomplete, missing, or wrong relevant information. Two important barriers regarding the social context were cultural problems (sense of awareness, but no broad support by everyone) and a lack of collaboration (i.e., not working together as a well-coordinated team). Frequent barriers at the level of the professional were defensive and disruptive attitudes and personal opinions toward the guidelines (i.e., finding the guidelines too excessive and the opinion that adherence to the guidelines may create a wrong sense of safety by weakening independent thinking and responsibility taking), behavioral routines that require adjustment, and a lack of awareness and knowledge about the importance and purpose of the guidelines. Perceived applicability of the stop moments during urgency care was the most important barrier at the patient level.

### Subgroup analyses

We found signals for possible differences in answers between perioperative key disciplines (see Additional file 3). Also, differences between types of hospital seemed to be present. For example, more respondents of peripheral hospitals than of academic or tertiary teaching hospitals seemed to think that measures are taken in their hospital to ensure that new employees are adequately instructed in the application of the perioperative guidelines. Respondents from tertiary teaching hospitals appeared to more often state to have an opinion leader/innovator within their discipline than respondents from academic or peripheral hospitals. Overall, subgroup analyses showed in 25% of the statements signals for differences between subgroups.

## Discussion

This nationally representative study identified 75 barriers to the success of the implementation of the perioperative safety guidelines, relating to time constraints, time pressure in emergency procedures, insufficient IT support and facilities, experienced increased workload, non-constructive attitudes and opinions toward the guidelines, lack of clinical documentation (i.e., missing, incomplete, or wrong information), suboptimal culture, lack of awareness, and knowledge about the importance and purpose, poor teamwork, lack of scientific evidence, complexity of the guidelines, lack of exposure to implementation efforts, suboptimal organizational preconditions, and lack of motivation to change behavioral routines. Several barriers already identified [19, 27, 29–32] were confirmed; these included a lack of time, inappropriateness in certain contexts such as emergencies, insufficient awareness, resisting opinions and insufficient knowledge of the professionals, inadequate IT infrastructures, social factors such as a suboptimal culture and collaboration (i.e., teamwork issues), skepticism regarding the evidence base, and inability to overcome the inertia of previous practice. However, we were not able to find other publications relating specifically to barriers concerning perioperative guidelines. Much research has been done on other guidelines and among other disciplines such as general practitioners, but the perioperative setting is underrepresented in implementation studies. Characterization of barriers and drivers to guideline implementation in this area is still needed.

The majority of the involved healthcare professionals seemed to agree with the specific guidelines and the concept of guidelines in general. Seventy-eight percent of the respondents felt (very) positive about guidelines in general. Other studies focusing on physicians’ attitudes toward guidelines demonstrated overall positive attitudes as well [33, 34]. Seventy-six percent believed that following the perioperative guidelines improves patient safety. Outcome expectancy is the expectation that a given behavior will lead to particular consequences; if a healthcare professional believes that a recommendation will lead to improved outcomes, adherence to this recommendation is more likely [35]. Motivation to work or start working according to the perioperative guidelines was valued at 7.5 on a scale of 10. Motivation of OR team members is considered essential for compliance with checklist use [36].

Only 56% of the respondents received regular feedback about perioperative incidents and complications that took place in their hospital. This might explain why only 51% of the responding healthcare professionals thought that the effects of using the guidelines are clearly visible. Furthermore, in line with previous literature [32], 42% considered a culture of audit and feedback as an important facilitator for the use of the perioperative safety guidelines.

It was remarkable that more than half of the respondents (54%) sometimes experienced (social) pressure not to work according to the perioperative guidelines and just one third (33%) perceived the relational culture of their hospital as open. In addition, only half of the respondents (51%) thought that everyone takes their responsibility in addressing each other in unsafe situations and guideline non-adherence. These issues often stem from a hierarchical team culture that obstructs the open culture and communication required to use the guidelines correctly. In almost all cases, an overly hierarchical culture is negatively associated with implementation of quality improvement and related practices. Riesenberg et al. [37] already reported communication barriers related to social structures and hierarchies in a study on nursing handoffs. To improve the safety culture, interventions should aim at minimizing this hierarchy [29]. The use of checklists is expected to improve communication among the OR team by the reduction of hierarchy barriers and unfamiliarity [38]. In particular, introducing every single team member by name and function before incision might create an atmosphere of mutual respect and acceptance and good team communication [39]. The existing hierarchies and the differential interprofessional status accorded to those in different disciplines must be altered in order to create psychological safety. Each member has to be allowed to take interpersonal risk by speaking up if any concern about safety arises without being afraid of being embarrassed, rejected, or punished [40, 41]. Psychological safety is a key antecedent of speaking up and learning behavior in healthcare teams [40].

In line with earlier literature [42], 42% of the respondents thought it was sometimes difficult to bring the entire team together to carry out a stop moment. Also, stop moments were performed when key professionals were absent or some staff were doing tasks away from the patient. This was also confirmed by the open responses in our study: “Doctors are not always on time or busy with several things at the same time (for example, looking at a patient on the ward). In order not to hamper the process, this will continue, without paying enough attention to the time-out. Sign-out agreements are almost always not discussed by the surgeon involved. OR assistants often spend time in the sterile preparation room during the time-out, so that none of them are present.”

“It remains difficult for some doctors to complete the questionnaire point by point with the whole team. Often, this is still a kind of a private chat. We do not always wait until the team is complete. Some colleagues continue with other ‘jobs’ during ‘topping’. In my opinion this should be a moment where everyone stops with what they are doing and finishes the TOP list as a team with full attention.”

Poor use of checklists has also the potential to deepen existing cultural division and further deteriorate interprofessional dynamics. When dominant team members decide to do the checks among themselves, other members of the team may feel excluded [43].

The most commonly mentioned barrier in the Barriers Top-3 was a lack of time. This was also confirmed by the results on the relevant barrier statement: only one third of the respondents (37%) experienced sufficient time to integrate the perioperative guidelines in their daily work. Consulting patients in an emergency situation was the next common barrier: 19% of the professionals perceived the guidelines to be inappropriate for time-pressured emergencies. These two barriers as well as workload are related to each other and have to do with work pressure and stress.

Time constraints are an external barrier. This may explain the discrepancy between the relative high motivation to work or start working according to the perioperative guidelines (a score of 7.5 out of 10) and actual guideline adherence in practice [10]. The conflict between time constraints as well as economic constraints and patient safety may also lead to increased stress for the personnel and negatively influence job satisfaction [44]. However, the performance of stop moments during urgent surgical procedures is not an external barrier and is under the healthcare professionals’ control. This barrier has to do with staff attitude and personnel’s conceptions of time and patient safety. The time investment of a stop moment in case of urgency is experienced as wasted time by many healthcare professionals. However, it has never been proven that the time spent conducting the stop moments takes too long in such a situation, and the duration of an average time-out procedure (1 to 3 minutes) [unpublished observation] does not confirm this either. Moreover, stops 1 and 3 do not apply in case of urgency, but this should not affect the execution of the other stop moments in the perioperative trajectory of emergency patients. Especially in urgent interventions, the risk of errors and complications is greater. In these circumstances, the execution of the stop moments is considered even more important.

A methodological strength of this study is the basis of an existing theoretical framework from the field of implementation science [21] for the online questionnaire with supplementary open-ended questions in order to obtain open responses. In this way, we tried to avoid that the determinants examined were solely dependent on the researchers’ selection and allowed the full range of implementation determinants and experiences to be explored. Our in-depth analysis of barriers and drivers provides detailed and new information to develop multifaceted implementation strategies for improving the implementation process of the perioperative safety guidelines. To the best of our knowledge, this is the first overview of quantitative research on the barriers and facilitators regarding the implementation of the national perioperative safety guidelines (with a multicenter hospital sample). We acknowledge that much has been written about (barriers and facilitators toward) the implementation of safety interventions such as surgical safety checklists. However, we have to make a distinction here. Our study concerned barriers and drivers for the implementation of guidelines that cover the complete perioperative process. That is something else than a single intervention. Parts of these guidelines, such as the stop moments, can be translated into a single intervention as, for example, a checklist. However, our study took the implementation of the entire underlying perioperative safety guidelines into consideration. By including different perioperative key disciplines from different types and sizes of hospitals, covering a representative sample of nine hospitals with geographic spread to cover multiple regions across the Netherlands, we ensured a breadth of perspectives, increasing the generalizability of our research. This generalizability may even partly hold for other areas than the perioperative one; some of the barriers and facilitators probably do not only apply for guideline implementation in the perioperative setting, since similar barriers and drivers have been previously identified as determinants influencing the implementation of change in healthcare [19, 27,30, 31]. The themes that emerged from this research probably represent the determinants that play a role in the implementation of many change initiatives in healthcare and hence present a valuable learning opportunity. Moreover, our study showed that the barriers and drivers on single safety interventions (e.g., surgical safety checklist) [29, 32] and the perioperative safety guidelines resemble each other. The response rate to the questionnaire of 41% was quite good, although we still collected opinions from a relatively small sample of perioperative healthcare professionals. Moreover, the response rate showed a skewed distribution. Anesthesiologists were somewhat overrepresented, and ward nurses were less well represented. This may, on the other hand, limit the ability to generalize our findings. Future studies would do well to aim for larger sample sizes, as the number of included hospitals was also small compared with the overall number of Dutch hospitals. Our study had several other limitations. Using a purposive, stratified sampling strategy based on relevance may have resulted in capturing the professionals with the strongest views about the guidelines and who are passionate about sharing them, meaning that we may have missed some valuable feedback from those whose opinions are less pronounced. Or those with a positive attitude toward guidelines may be overrepresented in our sample. We could not access demographic data of the non-respondents for reasons of confidentiality and were therefore unable to analyze the representativeness of our respondents. A non-response analysis could have provided more insight in this issue, but was unfortunately not feasible in the present study. Moreover, no postal addresses or telephone numbers were available to us, so we could reach respondents only via professional mail. Therefore, we were unable to increase the response rate by using additional methods to reach out to potential respondents. Non-response bias may, however, not be as crucial in health professionals’ surveys as in surveys of the general population [45]. Healthcare professionals are as a group more homogeneous regarding knowledge, training, attitudes, and behavior than the general population [45]. In previous studies analyzing non-respondents of survey research, non-response bias was suggested in research in which women, recently licensed, and younger medical specialists were more likely to respond [46, 47]. However, our study population consisted of a varied sample in terms of gender and experience. Furthermore, the reliability and validity of this non-validated survey is unknown. Replication of study results and analysis of questionnaire items is therefore warranted. Also, the perceived barriers depend on the situation-related perception of the healthcare professionals and may not accurately reflect the whole spectrum of barriers. Lastly, this analysis specified a list of barriers that were believed to influence implementation of the perioperative safety guidelines, but did not specify the interactions between them.

Implementation of new hospital processes is complex and requires careful evaluation and understanding of potential barriers and drivers. The results of this study provide input to improve the adherence to the Dutch national perioperative safety guidelines in practice. Besides, our findings may be useful to take into account in the process of updating these guidelines (to raise acceptance and implementability) as well as in the development and updates of other guidelines that need to be implemented in complex and high-risk environments in healthcare. These study results also emphasize that the implementation context (different hospital types and disciplines) seems to play some, but no overarching role in the identification of barriers toward the implementation of the perioperative safety guidelines.

The majority of health professionals in this study considered the presence of integrated information systems and audit and feedback as useful tools to support the use of the perioperative guidelines and stop moments. The implementation process of the perioperative guidelines could be enhanced by hospitals stimulating a culture of regularly feeding-back local data and results, in order to create learning experiences and insights in the effects of the guidelines. The regular provision of data and feedback would significantly increase buying into an intervention, particularly for those doubting its relevance, as reported by a qualitative evaluation of the barriers and facilitators to the implementation of the WHO surgical safety checklist across hospitals in England [32]. Therefore, it is important to focus on developing methods by which information on the impact of the guidelines can be captured, fed-back, and discussed periodically. Not doing so ultimately jeopardizes buy-in.

Future research should focus on identifying barriers at the recommendation level. For now, we only looked at the barriers that apply to the overall implementation process of the guidelines, not to individual guideline recommendations. A detailed analysis of the individual guideline recommendations may help in deciding where to focus the implementation efforts. Also, substantial improvements can be achieved by focusing on the barriers that are widely applicable across recommendations.

## Conclusions

The national perioperative safety guidelines were a safety improvement initiative required to be implemented in all Dutch hospitals. This study has identified multiple barriers for the implementation of these guidelines. According to our results, guideline adherence is affected by guideline factors, implementation factors, individual health professional factors, patient factors, and professional interactions, as well as by the (availability and acquisition of new) recourses and facilities and the capacity for organizational change. Reducing time pressure and workload, improving acceptance of the stop moments in urgency care, improving the IT structure, and regularly providing data and feedback regarding local benefits of the guidelines (e.g., data on reductions in complications and incidents, prevented near misses, or improved patient outcomes) are important to effectively improve the use of the perioperative safety guidelines and thereby improve perioperative patient safety. We have translated these findings into a number of recommendations that should be considered when implementing perioperative safety guidelines and are also relevant for changes in healthcare more broadly. To achieve reductions in work pressure and stress, it could be useful to make efficient alterations in the time allocation, increase staff or reconsider tasks and responsibilities, and improve IT facilities. By altering the perceived importance and perceptions about safe care and the time consumption involved in such a way that the stop moments make more sense in case of urgency, it is likely that compliance will increase. The perceived importance is strongly related to understanding the intentions and aims of the guidelines and stop moments in particular [29]. In addition, risk perception plays an important role [29]. Based on the results of this study, we suggest to give the perceived lack of applicability to emergency patients more prominent attention in implementation efforts (education and training of current staff and new employees). It is also important to focus efforts on developing methods by which the impact of the guidelines on daily performance can be captured and fed-back to staff. However, this needs a shift to a culture of intelligent data usage aimed at continuous quality and safety improvement. Data highlighting the local impact of the guidelines in terms of safer care can then be used by hospitals or disciplines to develop tailored and barrier-driven interventions to improve adherence in practice. This will also reinforce the personal relevance and interest in the guideline implementation. All this is probably what will motivate caregivers.

In conclusion, our study revealed a broad spectrum of barriers that perioperative key professionals perceive in applying a set of nationally developed perioperative safety guidelines. Implementation guidance should include explicit attention to these barriers and drivers. As a consequence, multiple interventions tailored to specific local barriers and drivers are needed to improve the use of the guidelines in practice. Results from this study help explain the suboptimal adherence in daily practice and provide useful suggestions for improving guideline adherence.

## Supplementary information

**Additional file 1.** PSIs for the pre-, per-, and postoperative care path.

**Additional file 2.** The answers on four open-ended questions.

**Additional file 3.** Summary of possible differences between the different professional groups and hospital types.

**Additional file 4.** The questionnaire survey.

## Data Availability

The dataset generated and analyzed during the current study is not publicly available as no informed consent for this has been obtained from the participating hospitals and individual professionals.

## Abbreviations

GGF: Green Wave Form
ICU: intensive care unit
IGJ: Inspectie Gezondheidszorg en Jeugd (Dutch Health Care Inspectorate)
IMPROVE: IMPlementatie Richtlijnen Operatieve VEiligheid (Implementation of Perioperative Safety Guidelines)
IT: Information technologies
MIDI: Measurement Instrument for Determinants of Innovations
OR: operation room
PSIs: Patient Safety Indicators
SD: Standard deviation
TNO: Dutch Organization for Applied Scientific Research
TOP: Toezicht Operatief Proces (Supervision of the Surgical Process)
WHO: World Health Organization
