# Supplementary material for: Reducing work pressure and IT problems and facilitating IT integration and audit & feedback help adherence to perioperative safety guidelines: a survey among 95 perioperative professionals
Source: Implement Sci Commun. 2020 May 27;1:49. doi: 10.1186/s43058-020-00037-1 (PMC7427904; doi:10.1186/s43058-020-00037-1)
Supplement: Supplementary file 1 — Additional file 1. PSIs for the pre-, per-, and postoperative care path. [file 43058_2020_37_MOESM1_ESM.docx]

Additional file 1: PSIs for the pre-, per- and postoperative care path

| N | Description | Type of indicator |
| --- | --- | --- |
| 1 | Completion of the total STOP-bundle, % patients  Seven separate stop moments in the perioperative care process:  I. Preoperative risk management – screening the patient  *The anesthesiologist assesses whether the perioperative risk is deemed acceptable by the patient, anesthesiologist, and surgeon, or appropriate measures are taken to minimize the risk and whether the patient agrees to the proposed operation, anesthetic treatment, and potential risks.*  II. Planning of the operation  *The operation date can be determined only when all conditions are met (i.e. agreements regarding preparation, peroperative details, and postoperative care). If not all conditions are met, this will be discussed and actions will be taken.*  III. Check of the current situation – checking whether all conditions for safe surgery are met  *Following the admission of the patient it will be verified whether all preoperative agreements have been carried out and no changes occurred in the condition of the patient. The surgeon is responsibility for this check.*  IV. Time-out before the operation  *Before the induction of anesthesia, a structural consultation takes place between surgeon, anesthesiologist, and OR staff; at least the following are discussed: correct patient, correct surgery, side/location, coagulation status, antibiotic policy, allergies, comorbidities, positioning of the patient, presence of skilled and qualified personnel, and appropriate materials. This consultation should take place in the operating room, before the start of anesthesia, and in presence of the patient. The surgeon is responsible for the performance and recording of this stop moment.*  V. Sign-out after the operation  *Before the patient leaves the operating room, a sign-out takes place in the operating room in the presence of the entire team. At the very least, the following are discussed and recorded: essential aspects of the procedure performed, count of the materials, and agreements relating to postoperative care. The surgeon is responsible for the performance and recording of this stop moment.*  VI. Discharge from the recovery  *Before the patient leaves the recovery room, the condition of the patient is checked using predetermined discharge criteria. The anesthesiologist is responsible for the performance and recording of this stop moment. The patient can only be discharged when predetermined criteria are met.*  VII. Discharge from the hospital  *Before the patient leaves the hospital, the clinical condition of the patient is checked. The patient can be discharged only if: - the medical condition of the patient permits this - care providers at the place of destination is are capable of safe and adequate treatment.*  *In the patient file is recorded: the decision to discharge the patient and when and by whom this decision is taken. The surgeon is responsible for the performance and recording of this stop moment.* | Process |
| 2A | Availability of a protocol on antibiotics (4 items) | Structure |
| 2B | Timely administration of antibiotic prophylaxis (% patients) | Process |
| 3 | Availability of a protocol on anticoagulants (5 items) | Structure |
| 4 | Availability of a protocol on responsibilities regarding maintenance of medical equipment (2 items) | Structure |
| 5 | Availability of a protocol on (performing) prospective risk analysis of medical equipment (8 items) | Process |
| 6 | Availability of OR regulations (7 items) | Structure |
| 7A | Presence of a surveillance system for postoperative wound infections (3 items) | Structure |
| 7B | Postoperative wound infections (in-hospital) (%) | Outcome |
| 8A | Presence of a morbidity and mortality registration (4 items) | Structure |
| 8B | Postoperative mortality (in-hospital) (%) | Outcome |
