# Supplementary material for: Reducing work pressure and IT problems and facilitating IT integration and audit & feedback help adherence to perioperative safety guidelines: a survey among 95 perioperative professionals
Source: Implement Sci Commun. 2020 May 27;1:49. doi: 10.1186/s43058-020-00037-1 (PMC7427904; doi:10.1186/s43058-020-00037-1)
Supplement: Supplementary file 2 — Additional file 2. The answers on four open-ended questions. [file 43058_2020_37_MOESM2_ESM.docx]

Additional file 2: The answers on four open-ended questions

| Question | **Is the execution of the stop moments fully integrated in the daily routines of the perioperative process within your hospital?** |
| --- | --- |
| Answers | *“Specialists want to operate certain patients even though the TOP is not 100% completed. Then they quickly make the surgery semi-urgent so that a patient does not need to have a 100% score.”*  *“In the meantime they have been fully integrated, but this does not mean that the guidelines are exactly followed during the execution of the various stop moments.”*  *“Too little awareness of the usefulness and necessity and there is no open contact culture whereby people get away with it.”*  *“Not all stop moments are useful.”*  *“I do not think that will ever work out completely, at least, I would not know how.”*  *“It has been realized that working according to the TOP 1, 2, and 3 increases the quality and safety for colleagues and patients. The supervision of the IGJ also contributed to this.”*  *“Partially. There is no full commitment yet. Agreements on how to act in case of a missed stop moments are lacking.”*  *“Nonchalance of certain people, but it gets better and the majority will certainly stick to it.”*  *“There is no routine yet.”*  *“When it stands in the way of efficient working, for example a too slow computer or overcrowding at the computer, it is not applied.”* |
| Question | **What are further obstacles for the use of the perioperative guidelines?** |
| Answers | *“There is always tension between time and money and the implementation of guidelines.”*  *“Some rules (such as no longer laying down medication and materials for the next patient while the previous one is still in the room) create unsafe situations. Now, after the time-out, the syringes are opened and medication is raised, fast, fast, double check. This happens no longer at a quiet time point, which seems to me really much safer!! I think that patient safety should be more important than a false sense of hygiene!!”*  *“Time, there is a chronic lack of it.”*  *“Time/production pressure, hierarchy (daring and willing), taking responsibility, and actions (sanctions).”*  *“Time pressure (to run production). Collaboration; there are too many islands that are busy with their own small part, but nobody is in control. A multitude of systems to work with.”*  *“Time pressure: production goes before everything.”*  *“Loss of time leads to loss of production, which leads to worse patient care (i.e. planning fewer procedures per session, so longer waiting times until necessary interventions can be performed).”*  *“Little to no consequences when the stop moments are not carried out.”*  *“Not seeing the usefulness of many guidelines; for many guidelines there is no scientific argumentation and therefore no large support, and through all checklists the patient is forgotten and there is more emphasis on the list than on the patient.”*  *“Doctors are not always on time or are busy with several things at the same time (for example, looking at a patient on the ward). In order not to hamper the process, this will continue, without paying enough attention to the time-out. Sign-out agreements are almost always not discussed by the surgeon involved. OR assistants often spend time in the sterile preparation room during time-out, so that none of them is present.”*  *“It remains difficult for some doctors to complete the questionnaire point by point with the whole team. Often, this is still a kind of private chat. We do not always wait until the team is complete. Some colleagues continue with other ‘jobs’ during ‘topping’; in my opinion this should be a moment where everyone stops with what they are doing and finishes the TOP list as a team with full attention.”*  *“Team availability, including the anesthesiologist for the stop moments like the time- and sign-out.”*  *“An open contact culture. If a surgeon or anesthesiologist does not follow the guidelines properly, there are only a few colleagues who dare to speak up to them about this. This is a hierarchical problem.”*  *“The autonomous attitude of specialists who are part of the OR team makes it difficult to comply with the guidelines in some cases. They have all sorts of arguments not to participate. Addressability for this is in some cases very low.”*  *“Main culture in a department (‘we never did that before’).”*  *“Not everyone takes every stop moment seriously and therefore some parts are poorly carried out.”*  *“Overkill of checklists and questions.”*  *“With the current focus on electronic registration in all areas, I notice that ‘record keeping must be correct on paper’ and the patient seems less important (‘Patient as Partner’??). Speaking with various healthcare professionals – medical, nursing and supportive – ‘registration fatigue’ is a very often-heard complaint.”*  *“A patient may appear for the anesthetic screening without a ‘Green Wave Form’ (GGF) being created. As a result, the anesthetic assessment cannot be filled out. This must be structurally different. A patient should not receive an appointment without a GGF.”*  *“We have the policy of safety and an open dialogue, but in practice they go for production.”* |
| Question | **What can we do to facilitate the implementation of the perioperative guidelines and stop moments?** |
| Answers | *“The only thing I have trouble with is not being allowed to prepare medication for the next patient. As a result, the patient is alone during the most important /exciting moment of the surgery, just before the induction of the anesthesia. This also leads to time pressure during the induction and emergence from general anesthesia, the most crucial moments of the anesthetic treatment.”*  *“Building smaller hospitals, with short lines, create organizations where people know each other again.”*  *“Simplify, everything digital (via computer) and not both paperwork and a digital version in some cases.”*  *“Provide employees with comprehensive information about the entire process so that there is more clarity about the whole. I think that if you get background information, you are more motivated to start new things.”*  *“In practice, it is sometimes very difficult to implement guidelines. We would like to show our own initiatives with regard to the manageability of the guidelines and let these be checked by the Inspectorate.”*  *“In my opinion, behavior and culture are the greatest obstacles, the problem has to be tackled there. I myself am a driver of the perioperative process, so I will have to continue to give the good example.”*  *“Make it fun.”*  *“A simpler procedure that requires less time and is more logical.”*  *“A digital option so you cannot leave the OR before everyone has signed (and so everyone must be/remain present).”*  *“Secure this more clearly. It should not only be a party of the Inspectorate or receive more attention because the Inspectorate can pay a visit. People need to be much more aware of the consequences of their actions.”*  *“Get rid of them.”* |
| Question | **Do you have any questions or comments, or do you want to add something?** |
| Answers | *“Maintaining guidelines and a lack of sanctions is, in my opinion, the biggest problem, just as culture and motivation.”*  *“Listen to the work floor more often and use clear communication.”*  *“A lot of people do not know the guidelines, even those who are supposed to work with them frequently.”*  *“Safety is great, but covering all risks for 100% must be weighed against the price (loss of efficiency). The TOP procedure undoubtedly takes time. I do not know a car driver who checks the engine and all the lights every time he drives a car. The fact that this happens with an airplane is indeed logical, but apparently we also accept that trains and ferries just leave without double checking everything. Why then with every routine intervention?”*  *“Often, I have the feeling that the guidelines are seen as a goal on their own. Not as a means of coming to a goal. When I have collected all signatures, I am ready. You cannot measure quality of care by this. To me, it is all about the patient. This is not the case anymore on the nursing wards. On the OR, this is different. Everything turns around the patient, literally and figuratively. But we have to record more and more. By doing this, we have to turn our back to the patient.”*  *“Visits of the Inspectorate and the threat of closure sped up all measures in a militaristic manner. This influenced the working climate negatively. This can be done better, more friendly, and more scientifically. Improper measures work counterproductively.”*  *“Retrospectively, I can say that working with the stop moments takes more time, but patient safety has improved enormously.”* |
