# Supplementary material for: Reducing work pressure and IT problems and facilitating IT integration and audit & feedback help adherence to perioperative safety guidelines: a survey among 95 perioperative professionals
Source: Implement Sci Commun. 2020 May 27;1:49. doi: 10.1186/s43058-020-00037-1 (PMC7427904; doi:10.1186/s43058-020-00037-1)
Supplement: Supplementary file 3 — Additional file 3. Summary of possible differences between the different professional groups and hospital types. [file 43058_2020_37_MOESM3_ESM.docx]

Additional file 3: Summary of possible differences between the different professional groups and hospital types

| **Question** | **Differences in answers**  (p<.05 threshold) |
| --- | --- |
| I agree with the content of the perioperative guidelines | Surgeon > Anesthesiologist  Ward nurse > Anesthesiologist |
| The perioperative guidelines are based on sufficient scientific evidence | ICU employee > Anesthesiologist  Ward nurse > Anesthesiologist |
|  |  |
| Working according to the perioperative guidelines goes at the expense of production | Surgeon > ICU employee  Anesthesiologist > ICU employee |
| The stop moments are sometimes skipped, while the patient continues to the next stage in the perioperative process | Anesthesia nurse > Surgeon |
| Working according the perioperative guidelines is beneficial | Ward nurse > Surgeon  Ward nurse > Anesthesiologist |
| The perioperative guidelines leave enough room for personal interpretation and adaptation to the specific needs of a department/discipline | Anesthesia nurse > Anesthesiologist  OR nurse > Anesthesiologist |
| I sometimes experience (social) pressure to work not according to the perioperative guidelines | Surgeon > Ward nurse  Anesthesiologist > Ward nurse  Anesthesia nurse > Ward nurse  OR nurse > Ward nurse  Recovery nurse > Ward nurse |
| In our hospital, we receive regular feedback on perioperative incidents and complications | Academic > Tertiary teaching |
| In our hospital, measures are taken to ensure that new employees are adequately instructed in the application of the perioperative guidelines | Peripheral > Academic  Peripheral > Tertiary teaching |
| In our hospital, there are enough financial resources available to apply the perioperative guidelines as intended | Peripheral > Tertiary teaching |
| Applying the perioperative guidelines leads to more workload | Academic > Peripheral |
| It is difficult to bring the entire team together for the execution of a stop moment, such as, for example, the time-out or sign-out | Tertiary teaching > Academic |
| The fact that security is high on the social agenda affects the use of the perioperative guidelines | Academic > Tertiary teaching |
| There is an opinion leader/innovator within my discipline | Tertiary teaching > Academic  Tertiary teaching > Peripheral |
