# Supplementary material for: Reducing work pressure and IT problems and facilitating IT integration and audit & feedback help adherence to perioperative safety guidelines: a survey among 95 perioperative professionals
Source: Implement Sci Commun. 2020 May 27;1:49. doi: 10.1186/s43058-020-00037-1 (PMC7427904; doi:10.1186/s43058-020-00037-1)
Supplement: Supplementary file 4 — Additional file 4. The questionnaire survey. [file 43058_2020_37_MOESM4_ESM.doc]

**
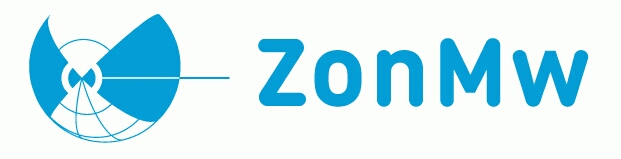

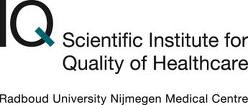

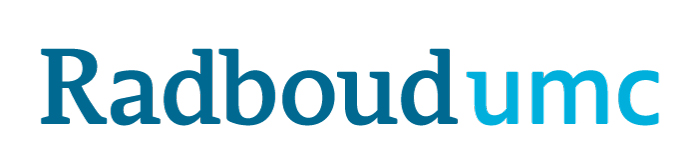
**

**IMPlementatie Richtlijn Operatieve VEiligheid**

**Contact:**

Yvette Emond, Radboud universitair medisch centrum, IQ Scientific Institute for Quality of Healthcare | Anesthesiologie, Pijn-en Palliatieve Geneeskunde, Postadres: IQ healthcare 114, Postbus 9101, 6500 HB Nijmegen, Telefoon: 024 36 67 311, Email: [yvette.emond@radboudumc.nl](mailto:y.emond@iq.umcn.nl)

**Barrièrevragenlijst IMPROVE**

|  | **In te vullen:** |
| --- | --- |
| **Ziekenhuis** |  |
| **Functie** | Chirurg  Anesthesioloog  Anesthesiemedewerker  Operatie‐assistent  Verkoever medewerker  IC medewerker  Verpleegkundige afdeling buik- en/of vaatchirurgie  Anders, namelijk |
| **Geslacht** | Man  Vrouw |
| **Werkervaring in jaren (incl. opleiding) in deze functie** | Totaal aantal jaren:       jaar  Aantal jaren in dit ziekenhuis:       jaar |
| **Datum** | (dd)-     (mm)-     (jjjj) |

**Toelichting:**

- Deze vragenlijst is ontwikkeld in het kader van de IMPROVE studie door drs. Yvette Emond en collega’s, afdelingen IQ healthcare en Anesthesiologie, Pijn-en Palliatieve Geneeskunde, Radboud universitair medisch centrum.

IMPROVE staat voor ‘IMPlementatie Richtlijnen Operatieve VEiligheid’. Deze drie richtlijnen geven aanbevelingen voor het preoperatieve, peroperatieve en het postoperatieve zorgtraject. De aanleiding voor de ontwikkeling van deze multidisciplinaire evidence-based richtlijnen waren de bevindingen van de Inspectie voor de Gezondheidszorg (IGZ) in hun TOP 1, 2 en 3 rapporten. De aanbevelingen in de richtlijnen betreffen niet de inhoud van het perioperatieve proces, maar het verloop van dit zorgproces. Verantwoordelijkheden zijn belegd, de aanpak van overdrachten en risicomomenten is gestandaardiseerd en stopmomenten worden gebruikt om te controleren of de gemaakte afspraken zijn nagekomen. Deze perioperatieve richtlijnen dienen geïmplementeerd te worden in alle Nederlandse ziekenhuizen. De IGZ controleert hier ook op.

- Het Radboudumc voert momenteel samen met o.a. uw ziekenhuis een onderzoek uit naar de implementatie van deze nationale perioperatieve richtlijnen.
- Het doel van deze vragenlijst is het in kaart brengen van de belemmerende en bevorderende factoren voor het toepassen van de **preoperatieve, peroperatieve en postoperatieve ('TOP 1, 2 en 3') richtlijnen, hierna genoemd de perioperatieve richtlijnen.**
- Het is belangrijk voor het onderzoek dat u **minimaal 1 jaar werkzaam bent in uw huidig ziekenhuis en bekend bent met de perioperatieve richtlijnen**. Bent u dit niet, verzoeken wij u de vragenlijst aan een collega door te geven die al langer werkzaam is in uw ziekenhuis en bekend is met de perioperatieve richtlijnen.
- Vragen die niet van toepassing zijn op uw werkzaamheden kunt u openlaten/overslaan.
- Het invullen van de vragenlijst neemt ongeveer **15-20 minuten** in beslag.
- Alle gegevens zullen **strikt vertrouwelijk** door ons worden behandeld en verwerkt. De gegevens worden alleen voor dit onderzoek gebruikt.
- Zou u de ingevulde vragenlijst indien mogelijk binnen 7 dagen terug willen sturen? Dit kan via

e-mail: [Yvette.Emond@radboudumc.nl](mailto:Y.Emond@iq.umcn.nl) of naar:

Radboudumc

114 IQ healthcare, t.a.v. Yvette Emond

Postbus 9101

6500 HB Nijmegen

- Leest u de vragen en antwoorden door en geef een antwoord dat het beste met uw mening overeenkomt. Denk niet te lang na. Elk antwoord is goed als het uw mening weergeeft.
- Er zijn twee soorten vragen:
  1. Als u bij een vraag uit meerdere antwoorden kunt kiezen, **vink dan één enkel antwoord aan, tenzij** staat aangegeven dat meerdere antwoorden mogelijk zijn. **Als u van mening wilt veranderen, moet u nogmaals op het kruisje klikken om dit te verwijderen**. U kunt vervolgens het antwoord dat u had willen geven aanvinken.
  2. Er zijn ook open vragen waarbij u het antwoord kunt invullen op de grijze vakken.
- **Vergeet u niet de vragenlijst op te slaan**

Deze vragenlijst is in een Word document. **U kunt in dit document typen** en de het vervolgens opslaan zoals u gewend bent. Het is niet nodig de gehele vragenlijst in een keer in te vullen. U dient de vragenlijst tussentijds wel op te slaan, anders gaan uw antwoorden verloren. U kunt de volgende keer dan weer gewoon verder gaan waar u was gebleven en de vragenlijst afmaken. Indien u dit gemakkelijker vindt, kunt u de vragenlijst echter ook uitprinten en dan invullen.

- Bij de meeste vragen staat een code tussen haakjes. De code verwijst naar de bron van de vraag.
  1. Cabana et al. Why don't physicians follow clinical practice guidelines? A framework for improvement. *JAMA* 1999; 282: 1458-65.
  2. TNO rapport (TNO 2012 R10625) Ontwikkeling meetinstrument voor determinanten van innovaties (MIDI).
  3. Richtlijn Het Peroperatieve Traject Bijlagen. Bijlage 5 Het implementatieplan.

Richtlijn Het Postoperatieve Traject Bijlagen. Bijlage 5 Het implementatieplan.

- Deze vragenlijst is gebaseerd op het theoretische model voor de implementatie van richtlijnen en innovaties van Van Sluisveld et al. (2013) en meet mogelijke barrières op 7 niveaus: interventie kenmerken, kenmerken van de professional, patiëntkenmerken, de sociale context, kenmerken van de organisatie, de samenleving en implementatie kenmerken.

**Alvast hartelijk dank voor uw medewerking!**

Werken volgens de perioperatieve richtlijnen pre-, per- en postoperatief traject

*Determinanten m.b.t. de innovatie (de perioperatieve richtlijnen)*

1. In hoeverre bent u op de hoogte van de inhoud van de perioperatieve richtlijnen? [1&2]

Ik ken de perioperatieve richtlijnen niet 🡪 *geef de vragenlijst door aan een collega*

Ik ken de perioperatieve richtlijnen wel, maar heb ze (nog) niet doorgelezen 🡪 *geef de vragenlijst door aan een collega*

Ik ken de perioperatieve richtlijnen en heb ze oppervlakkig doorgelezen/ bestudeerd

Ik ken de perioperatieve richtlijnen en heb ze volledig en grondig gelezen/ bestudeerd

1. Bent u het eens met de inhoud van de perioperatieve richtlijnen? [1]

Helemaal mee oneens

Mee oneens

Noch mee oneens, noch mee eens

Mee eens

Helemaal mee eens

1. Wat vindt u van het wetenschappelijk bewijs waarop de perioperatieve richtlijnen gebaseerd zijn? [1&2]

Ruim onvoldoende

Onvoldoende

Matig

Voldoende

Ruim voldoende

1. Wat vindt u van de begrijpelijkheid van de perioperatieve richtlijnen?

De perioperatieve richtlijnen geven helder aan welke activiteiten ik in welke volgorde moet uitvoeren. [1&2]

Helemaal mee oneens

Mee oneens

Noch mee oneens, noch mee eens

Mee eens

Helemaal mee eens

1. Wat vindt u van de compleetheid van de perioperatieve richtlijnen?

De perioperatieve richtlijnen bieden alle informatie en materialen die nodig zijn om er goed mee te kunnen werken. [2]

Helemaal mee oneens

Mee oneens

Noch mee oneens, noch mee eens

Mee eens

Helemaal mee eens

1. Wat vindt u van de complexiteit en uitvoerbaarheid van de perioperatieve richtlijnen? Zijn de richtlijnen moeilijk toe te passen?

De perioperatieve richtlijnen zijn te ingewikkeld voor mij om te kunnen gebruiken. [1&2]

Helemaal mee oneens

Mee oneens

Noch mee oneens, noch mee eens

Mee eens

Helemaal mee eens

1. Hoe passen de perioperatieve richtlijnen binnen uw werkwijze?

De perioperatieve richtlijnen sluiten goed aan bij hoe ik gewend ben om te werken. [1&2]

Helemaal mee oneens

Mee oneens

Noch mee oneens, noch mee eens

Mee eens

Helemaal mee eens

1. Vindt of vond u het moeilijk om uw dagelijkse routines en manier van werken aan te passen? [3]

Zeer zeker niet

Zeker niet

Soms niet, soms wel

Zeker wel

Zeer zeker wel

N.v.t., de perioperatieve richtlijnen sluiten goed aan bij hoe ik gewend was te werken

1. Vindt u dat de perioperatieve richtlijnen genoeg ruimte laten voor eigen invulling en aanpassing naar de specifieke behoeften van een afdeling/discipline? [2]

Veel te weinig

Te weinig

Genoeg

Te veel

Veel te veel

Weet ik niet

1. Ik vind de effecten van het gebruik van de perioperatieve richtlijnen duidelijk zichtbaar. [2]

Helemaal mee oneens

Mee oneens

Noch mee oneens, noch mee eens

Mee eens

Helemaal mee eens

1. Hoe staat u tegenover richtlijnen in het algemeen? [1]

Heel negatief

Negatief

Neutraal

Positief

Heel positief

*Determinanten m.b.t. de gebruiker (individuele professional)*

1. Ik vind het belangrijk om met behulp van de perioperatieve richtlijnen de patiëntveiligheid te verbeteren. [2]

Helemaal mee oneens

Mee oneens

Noch mee oneens, noch mee eens

Mee eens

Helemaal mee eens

1. Denkt u dat de perioperatieve richtlijnen de patiëntveiligheid zullen vergroten?

Ik verwacht dat met de perioperatieve richtlijnen de patiëntveiligheid wordt vergroot. [1&2]

Zeer zeker niet

Zeker niet

Misschien niet, misschien wel

Zeker wel

Zeer zeker wel

1. Vindt u dat er sprake was/is van een perioperatief veiligheidsprobleem in uw ziekenhuis? [3]

Zeer zeker niet

Zeker niet

Misschien niet, misschien wel

Zeker wel

Zeer zeker wel

1. Hoeveel wordt er in uw ziekenhuis, buiten de invoer van de perioperatieve richtlijnen, gedaan aan het verbeteren van patiëntveiligheid?

Zeer weinig

Weinig

Niet weinig, niet veel

Veel

Zeer veel

1. Sluiten de perioperatieve richtlijnen aan bij huidige of eerdere initiatieven op het gebied van patiëntveiligheid binnen uw ziekenhuis?

Zeer zeker niet

Zeker niet

Soms niet, soms wel

Zeker wel

Zeer zeker wel

N.v.t., geen andere initiatieven

1. Indien u dat wilt, lukt het u om volgens de perioperatieve richtlijnen te werken? Ook als u het druk heeft of als uw collega's het niet doen? [1&2]

Zeer zeker niet

Zeker niet

Soms niet, soms wel

Zeker wel

Zeer zeker wel

1. Ik beschik over voldoende kennis om de perioperatieve richtlijnen te kunnen gebruiken. [2&3]

Helemaal mee oneens

Mee oneens

Noch mee oneens, noch mee eens

Mee eens

Helemaal mee eens

1. Zijn de taken en verantwoordelijkheden rondom de perioperatieve richtlijnen bekend bij alle medewerkers? [2&3]

Zeer zeker niet

Zeker niet

Misschien niet, misschien wel

Zeker wel

Zeer zeker wel

1. Is iedereen het eens met de verdeling van taken en verantwoordelijkheden rondom de perioperatieve zorg?

Zeer zeker niet

Zeker niet

Sommigen niet, sommigen wel

Zeker wel

Zeer zeker wel

1. Het werken volgens de perioperatieve richtlijnen leidt tot een grotere werkdruk. [3]

Zeer zeker niet

Zeker niet

Soms niet, soms wel

Zeker wel

Zeer zeker wel

1. Het werken volgens de perioperatieve richtlijnen kost veel tijd, die ten koste gaat van de productie. [3]

Zeer zeker niet

Zeker niet

Soms niet, soms wel

Zeker wel

Zeer zeker wel

1. Het werken volgens de perioperatieve richtlijnen kost veel tijd, die ten koste gaat van de patiënten. [3]

Zeer zeker niet

Zeker niet

Soms niet, soms wel

Zeker wel

Zeer zeker wel

1. Vindt u dat het werken volgens de perioperatieve richtlijnen (genoeg) voordeel oplevert?

Zeer zeker niet

Zeker niet

Soms niet, soms wel

Zeker wel

Zeer zeker wel

1. Vindt u dat het werken volgens de richtlijnen uw klinische vrijheid/autonomie aantast? [3]

Zeer zeker niet

Zeker niet

Soms niet, soms wel

Zeker wel

Zeer zeker wel

*Patiëntgerelateerde determinanten*

1. In hoeverre verwachten patiënten dat u de perioperatieve richtlijnen toepast? [1&2]

Zeer zeker niet

Zeker niet

Misschien niet, misschien wel

Zeker wel

Zeer zeker wel

1. Als het gaat om het gebruik van de perioperatieve richtlijnen, hoeveel trekt u zich dan aan of zou u zich aantrekken van de mening van uw patiënten? [2]

Zeer weinig

Weinig

Niet weinig, niet veel

Veel

Zeer veel

*Determinanten m.b.t. de sociale setting en omgeving*

1. Geven uw collega's het goede voorbeeld door volgens de perioperatieve richtlijnen te werken? [3]

Zeer zeker niet

Zeker niet

Sommigen niet, sommigen wel

Zeker wel

Zeer zeker wel

1. Geven uw direct leidinggevenden het goede voorbeeld door volgens de perioperatieve richtlijnen te werken? [3]

Zeer zeker niet

Zeker niet

Sommigen niet, sommigen wel

Zeker wel

Zeer zeker wel

1. Hoeveel zorgverleners voor wie de perioperatieve richtlijnen zijn bedoeld, schat u, werken ook daadwerkelijk volgens de perioperatieve richtlijnen in uw ziekenhuis? [2]

Geen enkele zorgverlener

Bijna geen enkele zorgverlener

Een minderheid

De helft

Een meerderheid

Bijna alle zorgverleners

Alle zorgverleners

1. In hoeverre verwachten uw collega's dat u de perioperatieve richtlijnen toepast? [2]

Zeer zeker niet

Zeker niet

Misschien niet, misschien wel

Zeker wel

Zeer zeker wel

1. In hoeverre verwachten uw direct leidinggevenden dat u de perioperatieve richtlijnen toepast? [2]

Zeer zeker niet

Zeker niet

Misschien niet, misschien wel

Zeker wel

Zeer zeker wel

1. Als het gaat om het gebruik van de perioperatieve richtlijnen, hoeveel trekt u zich dan aan van de mening van uw collega's? [2]

Zeer weinig

Weinig

Niet weinig, niet veel

Veel

Zeer veel

1. Als het gaat om het gebruik van de perioperatieve richtlijnen, hoeveel trekt u zich dan aan van de mening van uw direct leidinggevenden? [2]

Zeer weinig

Weinig

Niet weinig, niet veel

Veel

Zeer veel

1. Ervaart u ook wel eens (sociale) druk om niet volgens de perioperatieve richtlijnen te werken?

Nooit

Soms

Vaak

1. Is het moeilijk om het hele team bij elkaar te brengen voor het uitvoeren van een stopmoment, zoals bijvoorbeeld de time-out of sign-out? [3]

Zeer zeker niet

Zeker niet

Soms niet, soms wel

Zeker wel

Zeer zeker wel

1. Vindt u dat er binnen uw discipline genoeg draagvlak is voor het werken volgens de perioperatieve richtlijnen?

Ruim onvoldoende

Onvoldoende

Matig

Voldoende

Ruim voldoende

1. Wilt u een rapportcijfer geven aan de motivatie/bereidheid binnen uw discipline om volgens de perioperatieve richtlijnen te (gaan) werken? [3]

*Van 1 t/m 10, waarbij geldt:*

*1= mijn discipline is helemaal niet gemotiveerd en bereid om volgens de perioperatieve richtlijnen te (gaan) werken en de stopmomenten uit te voeren*

*10= mijn discipline is volledig gemotiveerd en bereid om volgens de perioperatieve richtlijnen te (gaan) werken en de stopmomenten uit te voeren*

Rapportcijfer:

1. Hoe is de samenwerking met andere disciplines/tussen afdelingen ten aanzien van naleving van de perioperatieve richtlijnen? [2]

Zeer slecht

Slecht

Matig

Goed

Zeer goed

1. Ik kan op voldoende steun en betrokkenheid van het management rekenen bij het werken volgens de perioperatieve richtlijnen. [2&3]

Helemaal mee oneens

Mee oneens

Noch mee oneens, noch mee eens

Mee eens

Helemaal mee eens

1. Vindt u dat iedereen zijn verantwoordelijkheid neemt als het gaat om veilig werken; dus elkaar daadwerkelijk aanspreekt op onveilige situaties en het niet werken volgens de perioperatieve richtlijnen? [3]

Ruim onvoldoende

Onvoldoende

Matig

Voldoende

Ruim voldoende

1. In mijn ziekenhuis is er sprake van een open aanspreekcultuur, waarin iedereen elkaar durft aan te spreken op zijn of haar handelen en gedrag en dit ook door iedereen geaccepteerd wordt. [3]

Helemaal mee oneens

Mee oneens

Noch mee oneens, noch mee eens

Mee eens

Helemaal mee eens

*Determinanten m.b.t. de organisatie (organisatorische factoren) en economische context*

1. Op welke manier wordt er binnen uw ziekenhuis gecheckt of er volgens de perioperatieve richtlijnen gewerkt wordt?

Er wordt niet gecheckt

Er wordt wel gecheckt, namelijk als volgt:

1. Worden er maatregelen getroffen als er niet volgens de perioperatieve richtlijnen wordt gewerkt? Zijn er consequenties? Volgen er sancties?

Nee

Ja, namelijk:

1. In mijn ziekenhuis vindt regelmatig terugkoppeling plaats over incidenten en complicaties die hebben plaatsgevonden binnen de perioperatieve zorg in het ziekenhuis. [3]

Helemaal mee oneens

Mee oneens

Noch mee oneens, noch mee eens

Mee eens

Helemaal mee eens

1. Zijn er door het management in uw ziekenhuis formeel afspraken vastgelegd over het gebruik van de perioperatieve richtlijnen? (Bijv. in beleidsplannen, werkplannen en dergelijke) [2]

Nee

Ja

Weet ik niet

1. In mijn ziekenhuis zijn maatregelen getroffen zodat nieuwe medewerkers voldoende worden/zijn ingewerkt in het toepassen van de perioperatieve richtlijnen. [2]

Helemaal mee oneens

Mee oneens

Noch mee oneens, noch mee eens

Mee eens

Helemaal mee eens

1. Ik heb in mijn ziekenhuis gemakkelijk toegang tot informatie over het gebruik van de perioperatieve richtlijnen. [2]

Helemaal mee oneens

Mee oneens

Noch mee oneens, noch mee eens

Mee eens

Helemaal mee eens

1. Er is voldoende personeel/mankracht in mijn ziekenhuis om de perioperatieve richtlijnen zoals bedoeld te kunnen gebruiken. [1&2]

Helemaal mee oneens

Mee oneens

Noch mee oneens, noch mee eens

Mee eens

Helemaal mee eens

1. Er zijn voldoende financiële middelen/geld beschikbaar om de perioperatieve richtlijnen zoals bedoeld te kunnen gebruiken. [1&2]

Helemaal mee oneens

Mee oneens

Noch mee oneens, noch mee eens

Mee eens

Helemaal mee eens

1. Het ziekenhuis stelt mij voldoende tijd/uren beschikbaar om de perioperatieve richtlijnen zoals bedoeld te integreren in mijn dagelijks werk. [1&2&3]

Helemaal mee oneens

Mee oneens

Noch mee oneens, noch mee eens

Mee eens

Helemaal mee eens

1. Het ziekenhuis stelt mij voldoende materialen en voorzieningen beschikbaar om de perioperatieve richtlijnen zoals bedoeld te kunnen gebruiken. [2&3]

Helemaal mee oneens

Mee oneens

Noch mee oneens, noch mee eens

Mee eens

Helemaal mee eens

1. Welke middelen of methoden zouden nog ten goede komen aan de uitvoer van de perioperatieve richtlijnen c.q. stopmomenten? *Meerdere antwoorden mogelijk.* [1&2&3]

automatisering/geïntegreerde informatiesystemen

educatie

administratieve ondersteuning

(digitale) checklist

aanwezigheid van computers om de checklist in te vullen

indicatormetingen en feedback

geen

1. Wat zijn verdere belemmeringen bij het gebruik van de perioperatieve richtlijnen en waarom?

1. Hoe zijn deze belemmeringen ondervangen in uw ziekenhuis?

1. Worden de resultaten van het werken volgens de perioperatieve richtlijnen in termen van patiëntveiligheid (bijv. sterfte of complicaties) geëvalueerd? [3]

Nee

Ja

1. Krijgt u hiervan informatie teruggekoppeld? [3]

Nee

Ja

1. Zijn er, behalve de nieuwe perioperatieve richtlijnen, andere veranderingen waarmee u momenteel of binnen afzienbare tijd mee te maken heeft die mogelijk de invoering van de perioperatieve richtlijnen hinderen? (Bijv. reorganisatie, fusie, bezuinigingen, personeelsverloop, andere innovaties) [2]

Nee

Ja, namelijk:

*Determinanten m.b.t. de politieke en juridische context*

1. Is het feit dat veiligheid hoog op de maatschappelijke agenda staat voor u van invloed op het toepassen van de perioperatieve richtlijnen?

Zeer zeker niet

Zeker niet

Soms niet, soms wel

Zeker wel

Zeer zeker wel

1. Is een bezoek door de Inspectie voor de Gezondheidszorg voor uw ziekenhuis van invloed op het werken volgens de perioperatieve richtlijnen?

Zeer zeker niet

Zeker niet

Soms niet, soms wel

Zeker wel

Zeer zeker wel

Werken met de stopmomenten

1. Hebt u het idee dat in uw ziekenhuis het uitvoeren van de stopmomenten volledig geïntegreerd is in de dagelijkse routines binnen het perioperatieve proces?

Nee

Ja

*Indien nee/ja,* wat is hiervan denkt u de reden?

Hoe zou dit volgens u wel verwezenlijkt kunnen worden? Wat is hiervoor nodig of wat zou hiervoor moeten veranderen?

1. Worden stopmomenten ook weleens overgeslagen, terwijl de patiënt gewoon doorgaat naar de volgende fase binnen het perioperatieve traject? [3]

Nooit

Soms

Vaak

Locale implementatie van de perioperatieve richtlijnen in uw ziekenhuis

1. Is het voor u duidelijk wie de regie voert met betrekking tot de implementatie van de perioperatieve richtlijnen op uw afdeling?

Nee

Ja

Niemand voert de regie

1. Is er een voortrekker/opinieleider/innovator binnen uw discipline? [2&3]

Nee

Ja

1. Wordt er in uw ziekenhuis veel aandacht besteed aan de implementatie van de perioperatieve richtlijnen?

Zeer zeker niet

Zeker niet

Soms niet, soms wel

Zeker wel

Zeer zeker wel

1. Hoe wordt/werd kennis omtrent de perioperatieve richtlijnen en het uitvoeren van de stopmomenten uit de richtlijnen binnen uw discipline overgebracht? Welke hulpmiddelen/interventies worden/werden gebruikt om te zorgen dat de kennis bij iedereen terecht komt? (Bijv. training van de medewerkers, printen van de perioperatieve richtlijnen en verspreiden in de postvakjes)

1. Heeft u deelgenomen/gebruik gemaakt van deze interventies?

Nooit

Soms

Vaak

Altijd 🡪 *ga naar vraag 69*

1. Wat waren voor u redenen om niet altijd deel te nemen/gebruik te maken van de aangeboden interventies? *Meerdere antwoorden mogelijk.*

Tijdsdruk

Ik vond het overbodig / ik twijfelde over de meerwaarde van de interventies

Mijn collega's namen ook niet deel/maakten ook geen gebruik

Deelname/gebruik was vrijblijvend

Deelname/gebruik had voor mij geen prioriteit

Afwezigheid tijdens interventies

Anders, namelijk:

1. Wat vindt u van het aantal interventies die zijn ondernomen om de perioperatieve richtlijnen op de agenda te zetten binnen uw ziekenhuis?

Veel te weinig

Te weinig

Genoeg

Te veel

Veel te veel

1. Voelt u zich genoeg betrokken bij de implementatie van de perioperatieve richtlijnen in uw ziekenhuis? [2]

Ruim onvoldoende

Onvoldoende

Matig

Voldoende

Ruim voldoende

Barrièretop 3

1. Kunt u aangeven wat voor u de drie belangrijkste/grootste belemmeringen zijn voor het toepassen van de perioperative richtlijnen? (Dus redenen om niet (volledig) volgens de richtlijnen te werken)

1.

2.

3.

Toekomstige implementatie van de perioperatieve richtlijnen

1. Wat kunnen wij voor u betekenen om de implementatie van de perioperatieve richtlijnen en stopmomenten te faciliteren?

1. Hebt u nog vragen/opmerkingen of wilt u nog iets toevoegen?

**Hartelijk dank voor het invullen van de vragenlijst!**

**Vergeet u niet de vragenlijst op te slaan en zo spoedig mogelijk te retourneren?**
